# The Effect of the PCSK9 Inhibitor Evolocumab on Aldosterone Secretion among High Cardiovascular Risk Patients: A Pilot Study

**DOI:** 10.3390/jcm10112504

**Published:** 2021-06-05

**Authors:** Elena Izkhakov, Yacov Shacham, Merav Serebro, Iris Yaish, Yonit Marcus, Gabi Shefer, Karen Tordjman, Yona Greenman, Naftali Stern, Tomer Ziv-Baran

**Affiliations:** 1 Institute of Endocrinology, Metabolism and Hypertension, Tel Aviv Sourasky Medical Center, Tel Aviv 64239, Israel; meravs@tlvmc.gov.il (M.S.); irisy@tlvmc.gov.il (I.Y.); yonitm@tlvmc.gov.il (Y.M.); gabish@tlvmc.gov.il (G.S.); karent@tlvmc.gov.il (K.T.); yonagr@tlvmc.gov.il (Y.G.); naftalis@tlvmc.gov.il (N.S.); 2 Sackler Faculty of Medicine, Tel Aviv University, Tel Aviv 69978, Israel; kobys@tlvmc.gov.il; 3 Department of Cardiology, Tel Aviv Sourasky Medical Center, Tel Aviv 64239, Israel; 4 School of Public Health, Sackler Faculty of Medicine, Tel Aviv University, Tel Aviv 69978, Israel; zivtome@tauex.tau.ac.il

**Keywords:** PCSK9 inhibitors, aldosterone, cortisol, hypertension

## Abstract

Elevated low-density lipoprotein (LDL) cholesterol is one of the leading causes of cardiovascular disease. Proprotein convertase subtilisin/kexin type 9 (PCSK9) inhibitors reduce LDL cholesterol levels with subsequent reductions in cardiovascular morbidity. Elevated aldosterone levels are also associated with a greater risk of cardiovascular morbidity. There are currently no published data on the impact of PCSK9 inhibitor monotherapy on the secretion of aldosterone. The aim of this study was to examine the effect of monotherapy with the PSCK9 inhibitor evolocumab on the lipid profile and aldosterone secretion level in high-risk cardiovascular patients. Lipid profile, sodium, potassium, aldosterone, cortisol, plasma renin activity, and adrenocorticotropic hormone (ACTH) levels were analyzed at baseline and after 3 months of evolocumab therapy. Each participant underwent a 250 mcg ACTH stimulation test upon study entry. Eight women and seven men were included in the study. Their median total cholesterol, LDL cholesterol, lipoprotein (a), apolipoprotein B100, and baseline and stimulated aldosterone levels were significantly lower after 3 months of evolocumab therapy. These heretofore unreported findings indicate that reductions in unstimulated and stimulated aldosterone secretion under evolocumab therapy could be associated with reductions in cardiovascular events, a possibility that warrants further investigation.

## 1. Introduction

Adrenocortical and gonadal steroid hormones are derived from cholesterol. Low-density lipoprotein (LDL) particles are the major source of cholesterol for the purpose of steroid synthesis in both glands. High LDL cholesterol levels comprise one of the leading cardiovascular risk factors, and their reduction causes significant decreases in cardiovascular morbidity and mortality. Therefore, in situations of very low plasma levels of LDL cholesterol, the transportation of these particles into steroid-producing organs might hypothetically be reduced and possibly result in the impaired production of steroid hormones [1]. An example of this chain of events is abetalipoproteinemia, a rare inherited disease characterized by very low/absent apolipoprotein B (Apo B)-containing particles, including chylomicrons, very low lipoprotein (VLDL) and LDL [2]. This condition can be accompanied by a significant decrease in adrenocortical and gonadal function [3,4].

In 2015, the US Food and Drug Administration and the European Medicines Agency approved the use of two drugs from the new lipid-lowering group, the proprotein convertase subtilisin/kexin type 9 (PCSK9) inhibitors alirocumab and evolocumab. They were designated for patients with either familial hypercholesterolemia or clinical cardiovascular atherosclerosis, or patients at very high risk of atherosclerosis and who were not otherwise able to achieve the target values of LDL cholesterol.

PCSK9 inhibitors decrease the degradation of the LDL receptors located on the surface of hepatocytes, thereby increasing the removal of LDL particles from the blood and significantly reducing the values of LDL cholesterol by an average of 60% in individuals at high cardiovascular risk or those with established cardiovascular disease [5,6]. In addition to their LDL cholesterol-reducing effects, the non-lipid-lowering role of PCSK9 inhibitors in the improvement of endothelial function, the reduction of inflammatory markers, and platelet aggregation and activation has also been described [7]. Those drugs constitute a highly important addition to the primary and secondary prevention of cardiac sequelae among patients who have either failed to achieve the target values of LDL cholesterol with statins or other lipid-lowering drugs, or those who have sustained statin intolerance [8,9].

Aldosterone is a steroidal hormone whose elevation predicts cardiovascular morbidity and mortality [10]. To date, there are no published data on the impact of PCSK9 inhibitor monotherapy on aldosterone secretion.

The aim of this study, therefore, was to examine the effect of 3 months of monotherapy with the PCSK9 inhibitor evolocumab 140 mg administered subcutaneously once every 2 weeks on the lipid profile and aldosterone secretion levels in statin-intolerant men and women who were diagnosed as being at high cardiovascular risk.

## 2. Patients and Methods

This is an interventional pilot study of high-cardiovascular-risk patients who were unable to tolerate statins. They were being treated for dyslipidemia at the Institute of Endocrinology, Metabolism, and Hypertension at the Tel Aviv Sourasky Medical Center (TASMC).

Dyslipidemia was defined by at least two plasma low-density lipoprotein cholesterol measurements higher than 70 mg/dL. Diabetes mellitus was defined by at least one of the following: (1) an established diabetes mellitus diagnosis; (2) two fasting plasma glucose measurements higher than 125 mg/dL; (3) a random plasma glucose measurement higher than 199 mg/dL; (4) hemoglobin A1C levels of 6.5% or higher. Hypertension was defined by at least one of the following: (1) an established hypertension diagnosis; (2) three or more measurements of systolic blood pressure higher than 140 mmHg; (3) three or more measurements of diastolic blood pressure higher than 90 mmHg.

The study inclusion criteria were age over 18 years, and being at high cardiovascular risk with or without ischemic heart disease while on stable medical treatment during the preceding month and unable to tolerate statins.

Excluded were patients who had a previous history of any systemic inflammatory disease, statin use one month prior to study entry, pregnancy, breast-feeding, uncontrolled hypertension, and known malignancies within the 5 years preceding study entry.

The study was approved by the TASMC medical ethics committee (Institutional Board Review Number: TLV-15-0759). Written informed consent was obtained from all patients prior to enrollment. All procedures performed in studies involving human participants were in accordance with the ethical standards of the institution and with the 1964 Helsinki declaration.

### 2.1. Study Procedure

After recruitment into the study, the participants underwent a physical examination, height and weight measurements for body mass index determination, and blood pressure and heart rate measurements. Blood samples were taken between 08:00 and 09:00 after a 12 h fast for the assessment of glucose, lipid profile, sodium, potassium, aldosterone, cortisol, ACTH, lipoprotein (a) (Lp(a)), apolipoprotein B100 (Apo B100), and apolipoprotein A1 (Apo A1) levels, at both baseline and 3 months following therapy with subcutaneous evolocumab 140 mg once every 2 weeks. Each participant underwent a 250 mcg ACTH stimulation test and 24 h ambulatory blood pressure monitoring (ABPM) at the beginning and end of the study (3 months following evolocumab therapy). The participants were asked to continue with their normal daily activities, diet, and chronic medical therapy during the study period.

### 2.2. ACTH Stimulation Test

The ACTH stimulation test was performed in quiet surroundings, and after 30 min of absolute rest. ACTH 250 micrograms was injected intravenously, and blood samples for cortisol, aldosterone and plasma renin activity (PRA) were drawn at baseline and at 30 and 60 min after the injection.

### 2.3. Assays

Serum total cholesterol, HDL cholesterol, triglycerides, sodium, potassium, and glucose assays were measured using an enzymatic method (SIEMENS Advia 2400 Chemistry Analyzer). LDL cholesterol was calculated according to the Friedewald formula (LDL = total cholesterol–HDL cholesterol–triglycerides/5; mg/dL). Lp(a), Apo B100, and Apo A1 were determined by an immunoturbidimetry procedure (Abbott ARCHITECT *c*System). Serum total cortisol was measured by electrochemiluminescent assay (Cobas e411, ROCHE Diagnostic, Indianapolis, IN, USA), serum aldosterone by chemiluminescent immunoassay technology (LIAISON XL USA), PRA by ELISA competitive assay (IBL company, Minneapolis, MN, USA), and plasma ACTH by chemiluminescent assay (IMMULITE 2000XP SIEMENS Diagnostics, Manchester, UK).

### 2.4. Twenty-Four Hour Ambulatory Blood Pressure Monitoring

All patients underwent a 24 h ABPM with a fully automatic device (SpaceLabs 90207; Spacelabs Healthcare, Snoqualmie, WA, USA) at the beginning and end of the study. Blood pressure readings were obtained every 20 min during daytime and every 30 min during nighttime. The participants were asked to continue with their normal daily activities during measurements but refrain from movement during the measurement.

### 2.5. Statistical Analysis

Categorical variables were reported as frequency and percentage. Continuous variables were evaluated to meet normal distribution by means of a histogram and the Kolmogorov–Smirnov test. The distribution of each variable was skewed and therefore described as median and first and third quartile (Q1–Q3). The Wilcoxon signed-rank test was applied to compare the values of each variable at baseline and at the 3-month follow-up. All statistical tests were 2-sided and a *p*-value of <0.05 was considered statistically significant. All data were analyzed with SPSS software (IBM SPSS Statistics, version 25, IBM corp., Armonk, NY, USA, 2017).

## 3. Results

### 3.1. Characteristics of the Study Population

Fifteen patients (eight women and seven men, median age 63.8 years (Q1–Q3: 59.5–69.6)) were included in the study. The clinical characteristics of the study population at the beginning of the study are listed in Table 1. Nine of the participants were patients with known ischemic heart disease and six of them were high-cardiovascular-risk patients (Table 1). None of the 15 patients were able to tolerate statins due to adverse muscular effects. All participants completed the study. There were no side effects related to evolocumab use or the ACTH stimulating test.

### 3.2. Changes in Lipid Profile

Significantly lower levels of total cholesterol (by 38%, *p* = 0.001), LDL cholesterol (by 50%, *p* = 0.001), Lp(a) (by 17%, *p* = 0.002), and Apo B100 (by 43%, *p* = 0.002) were found 3 months following evolocumab therapy (Table 2). There were no significant changes in triglycerides and HDL cholesterol levels.

### 3.3. Changes in Steroidogenesis

**Figure 1:**
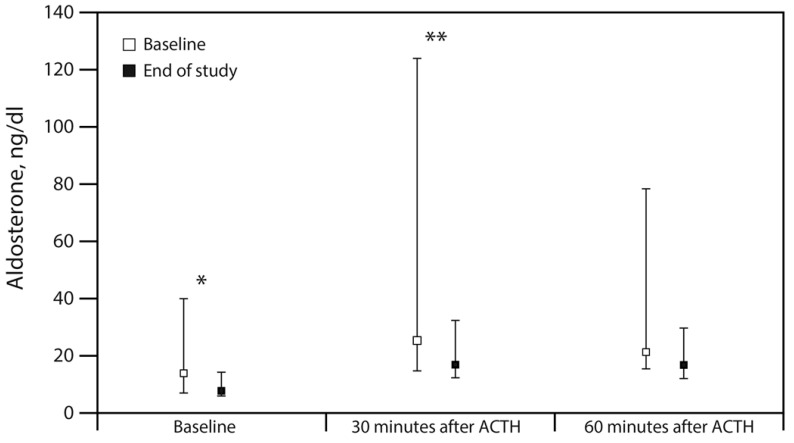
Aldosterone changes during the 250 mcg ACTH stimulation test at the beginning and end of the study. The distribution of aldosterone is shown as median and first and third quartile (Q1–Q3). ACTH indicates adrenocorticotropic hormone; * *p* < 0.05; ** *p* < 0.01.

The main finding of this study was the discovery that evolocumab decreases the amount of aldosterone secretion. Baseline aldosterone levels were reduced by more than 40% at 3 months into the evolocumab therapy (7.9 ng/dL, Q1–Q3: 5.6–14.5 compared to 13.9 ng/dL, Q1–Q3: 6.7–40.4, *p* = 0.036). Likewise, aldosterone levels rose significantly less after evolocumab therapy 30 min after ACTH stimulation (16.9, Q1–Q3: 12.0–32.6 vs. 25.2, Q1–Q3: 14.6–124.3, *p* = 0.008; Table 3, and Figure 1). In contrast, the cortisol level was unaffected, both in the basal state and after ACTH stimulation, and this was mirrored by the lack of alteration in plasma ACTH concentrations. Interestingly, this reduction in aldosterone was not accompanied by changes in either PRA or in serum potassium (Table 3).

### 3.4. Changes in 24-Hour ABPM

The night diastolic blood pressure levels were significantly lower (median difference 3 mmHg, Q1–Q3: 0.5–6.5; *p* = 0.03) at 3 months following evolocumab therapy. No additional significant blood pressure changes were detected.

## 4. Discussion

The results of this short-term intervention pilot study revealed that a 3-month evolocumab monotherapy was associated with reduced baseline and stimulated aldosterone levels, while PRA, cortisol and ACTH levels did not change significantly. These changes were accompanied by a slightly decreased night diastolic blood pressure. In addition, the monotherapy produced the classic effects on the lipid profile, i.e., reductions in total cholesterol, LDL cholesterol, Lp(a), and Apo B100. Dyslipidemia is one of the leading cardiovascular risk factors, and a significant reduction in LDL cholesterol is highly important for the prevention and treatment of atherosclerotic cardiovascular disease, including ischemic heart disease, cerebrovascular disease, and peripheral artery disease. Clinical and pathophysiological evidence has supported the safety of extremely low LDL levels with subsequent reductions in the development of cardiovascular disease [11]. Lp(a) is another independent cardiovascular risk factor associated with high cardiovascular morbidity and mortality, and lowering Lp(a) levels can be an independent contributor to MACE reduction in patients with acute coronary syndrome [12]. However, there is strong evidence that the renin angiotensin aldosterone system (RAAS) is an additional key contributor to the development and progression of cardiovascular disease, and that its activation should be reduced [13].

Statins (inhibitors of 3-hydroxy-3-methylglutaryl CoA reductase), the first-line treatment for dyslipidemia, decrease the levels of LDL cholesterol and significantly reduce the risk of the development and progression of atherosclerosis, cardiovascular morbidity, and mortality, as well as the need for re-vascularization [14]. Due to the significant reduction in cholesterol production caused by lipid-lowering drugs, questions arose about their possible effect on steroidogenesis. Earlier studies [15,16,17,18] reported relatively higher levels of LDL cholesterol (100–130 mg/dL) as therapeutic targets, but failed to convincingly confirm this potential in statins. However, a later meta-analysis by Schooling et al. [19] that integrated the results of 11 randomized controlled trials demonstrated a moderate but significant decline in the values of testosterone in patients treated with statins, among both men (a decline of 3.4%) and women (a decline of 12.6%). Baudrand et al. [20] showed that high doses of lipophilic statins decreased aldosterone secretion in response to angiotensin II and a low-salt diet in two human interventional studies. The mechanisms underlying this effect remain unclear. Finally, Toka et al. [21] demonstrated the suppression of the renal expression of mRNA for angiotensin-converting enzyme (ACE) and CYP11B2, aldosterone synthase, and the amount of aldosterone in the rat kidney following pitavastatin treatment.

It is worth noting that the transfer protein inhibitor cholesterol ester torcetrapib was removed from clinical practice due to increased mortality after it was revealed that it led to higher values of aldosterone [22], further supporting the significance of the negative effects of aldosterone on mortality among high-cardiovascular-risk patients.

Blom et al. investigated the effects of 52 weeks of monthly subcutaneous evolocumab treatment with one of the following: diet, atorvastatin 10 mg, atorvastatin 80 mg, and a combination of atorvastatin 80 mg and ezetimibe 10 mg [23]. Those authors found a minimal but significant reduction in the values of vitamin E in correlation with lower LDL cholesterol levels. They also found a slight increase in circulating cortisol. There were no changes in circulating ACTH, the ratio of cortisol:ACTH, total testosterone, or estradiol levels (aldosterone levels were not examined in that study).

The present study demonstrates a significant reduction in the lipid profile (total cholesterol, LDL cholesterol, Lp(a), and Apo B100), as well as in baseline and stimulated aldosterone values. These changes were accompanied by a slight but significant reduction in night diastolic blood pressure levels, without any specific effect on fasting blood glucose under evolocumab monotherapy. Two cardiovascular outcome studies [24,25] on treatment with the PSCK9 inhibitors alirocumab and evolocumab showed significant improvements in lipid profiles accompanied by reductions in cardiovascular morbidity and mortality. Taken together with the findings of the present study, it is reasonable to consider that aldosterone may also play a partial role in producing these effects. The mechanisms underlying these findings should be investigated in further studies.

The first limitation of this pilot study is the relatively small sample size. Second, it is a single-center and non-randomized study without a control group, with all participants serving as their own controls. Third, the follow-up period was relatively short, although it was long enough to observe post-treatment changes in the lipid profile, aldosterone secretion, and diastolic blood pressure.

In conclusion, this study provides heretofore unreported evidence that the inhibition of PCSK9 by evolocumab decreased unstimulated and stimulated aldosterone secretion, accompanied by a reduction in night diastolic blood pressure and by beneficial changes in the lipid profile. The reduction in aldosterone levels may be at least partially responsible for the positive effect of the inhibition of PCSK9 on cardiovascular morbidity and mortality. Such a possibility warrants prospective randomized studies, and an assessment of the mechanisms involved in aldosterone reduction by PCSK9 inhibitors as a monotherapy and in combination therapies with statins.

## Figures and Tables

**Table 1:** Baseline characteristics of the study population (*n* = 15).

Variable	Value
Median age, y (Q1–Q3)	63.8 (59.5–69.6)
Male, *n* (%)	7 (47)
Body mass index, median (Q1–Q3)	29.4 (27.7–33.9)
Weight, kg, median (Q1–Q3)	86.3 (69.5–97.2)
Hyperlipidemia, *n* (%)	15 (100)
Diabetes mellitus, *n* (%)	6 (40)
Hypertension, *n* (%)	9 (60)
Past myocardial infarction, *n* (%)	9 (60)
Smoking, *n* (%)	1 (7)
Family history of ischemic heart disease, *n* (%)	9 (60)

Abbreviations: Q1, first quartile; Q3, third quartile.

**Table 2:** Blood biochemistry data of the study population.

Parameter	Baseline Median (Q1–Q3)	End of Study Median (Q1–Q3)	Difference Median (Q1–Q3)	*p* Value
FBG, mg/dL	90 (80–107)	87 (79–98)	−3 (−14–10)	0.176
Potassium, mmol/L	4.4 (4.2–4.6)	4.4 (4.2–4.7)	0 (−0.6–0.3)	0.889
Total cholesterol, mg/dL	219 (156–247)	136 (118–162)	−55 (−99–−41)	0.001
HDL cholesterol, mg/dL	46 (37–64)	47 (38–59)	1 (−2–3)	0.706
LDL cholesterol, mg/dL	127 (93–153)	63 (43–73)	−65 (−92–−41)	0.001
Triglycerides, mg/dL	146 (97–190)	114 (101–181)	1 (−35–7)	0.496
Lipoprotein (a), mg/dL	66 (17–92)	52 (11–83)	−11 (−120–−5)	0.002
Apo A1, mg/dL	144 (123–172)	149 (135–158)	4 (−4–17)	0.505
Apo B100, mg/dL	117 (74–150)	65 (51–81)	−50 (−65–−13)	0.002

Abbreviations: FBG, fasting blood glucose; HDL, high-density lipoprotein; LDL, low-density lipoprotein; Apo A1, apolipoprotein A1; Apo B100, apolipoprotein B100; Q1, first quartile; Q3, third quartile.

**Table 3:** Results of the 250 mcg ACTH stimulation test.

Parameter	Baseline	End of Study	Difference	*p* Value
ACTH, pg/mL				
Baseline	17.6 (6.7–23.4)	18.0 (11.1–24.1)	0.1 (−4.1–5.9)	0.638
Cortisol, mcg/dL				
Baseline	13 (10.3–15.8)	12.3 (8.9–19.3)	0.1 (−2.5–1.7)	0.975
30 min	30 (25.2–32.4)	28.1 (23.9–37.2	0.9 (−2.6–4.3)	0.552
60 min	33.9 (29.9–38)	31.1 (28.4–41.1)	−0.1 (−2.4–3.1)	0.701
Aldosterone, ng/dL				
Baseline	13.9 (6.7–40.4)	7.9 (5.6–14.5)	−2.9 (−37.6–-0.3)	0.036
30 min	25.2 (14.6–124.3)	16.9 (12.0–32.6)	−3.4 (−116.3–−1.1)	0.008
60 min	21.3 (15.2–78.6)	16.8 (12.2–29.8)	3.9 (−36.5–1.1)	0.064
PRA, ng/mL/h				
Baseline	1.5 (0.8–3.6)	1.5 (0.8–3.6)	−0.2 (−1.0–0.6)	0.65
30 min	1.2 (0.3–3.8)	1.2 (0.3–3.8)	0.3 (−0.2–1.1)	0.328
60 min	0.8 (0.6–3)	0.8 (0.6–3)	−0.2 (−0.7–0.8)	0.972

Abbreviations: ACTH, adrenocorticotropic hormone; PRA, plasma renin activity; Q1, first quartile; Q3, third quartile.

## Data Availability

The data underlying this article will be shared on reasonable request to the corresponding author.
